# Biochemical Characterization and Elucidation of the Hybrid Action Mode of a New Psychrophilic and Cold-Tolerant Alginate Lyase for Efficient Preparation of Alginate Oligosaccharides

**DOI:** 10.3390/md20080506

**Published:** 2022-08-05

**Authors:** Shengsheng Cao, Li Li, Benwei Zhu, Zhong Yao

**Affiliations:** 1 College of Food Science and Light Industry, Nanjing Tech University, Nanjing 211816, China; 2 Suqian Advanced Materials Industry Technology Innovation Center of Nanjing Tech University, Suqian 223800, China

**Keywords:** alginate lyase, biochemical characterization, endo/exolytic, psychrophilic, cold-tolerant

## Abstract

Alginate lyases with unique biochemical properties have irreplaceable value in food and biotechnology industries. Herein, the first new hybrid action mode *Thalassotalea algicola*-derived alginate lyase gene (TAPL7A) with both psychrophilic and cold-tolerance was cloned and expressed heterologously in *E. coli*. With the highest sequence identity (43%) to the exolytic alginate lyase AlyA5 obtained from *Zobellia galactanivorans*, TAPL7A was identified as a new polysaccharide lyases family 7 (PL7) alginate lyase. TAPL7A has broad substrate tolerance with specific activities of 4186.1 U/mg, 2494.8 U/mg, 2314.9 U/mg for polyM, polyG, and sodium alginate, respectively. Biochemical characterization of TAPL7A showed optimal activity at 15 °C, pH 8.0. Interestingly, TAPL7A exhibits both extreme psychrophilic and cold tolerance, which other cold-adapted alginate lyase do not possess. In a wide range of 5–30 °C, the activity can reach 80–100%, and the residual activity of more than 70% can still be maintained after 1 h of incubation. Product analysis showed that TAPL7A adopts a hybrid endo/exo-mode on all three substrates. FPLC and ESI-MS confirmed that the final products of TAPL7A are oligosaccharides with degrees of polymerization (Dps) of 1–2. This study provides excellent alginate lyase candidates for low-temperature environmental applications in food, agriculture, medicine and other industries.

## 1. Introduction

Alginate is a structural polysaccharide in the cell wall of *Phaeophyta*, such as *Saccharin japonica* and *Undaria pinnatifida* [1]. Alginate consists of β-ᴅ-mannuronate (M) and its C5 epimer α-ʟ-guluronate (G) randomly combined by 1,4 glycosidic bonds [1]. Alginate has been widely used in food, agriculture, medicine, materials and other industrial fields because of its excellent properties. For example, alginates can be used as food stabilizers and thickeners to improve the stability of ice cream [2]. As the products of alginate lyase degradation of alginate, alginate oligosaccharides (AOS) have the advantages of outstanding solubility and high bio-availability under the same biochemical activity as polysaccharide. Alginate oligosaccharides have probiotic properties and can regulate the intestinal micro-ecological balance by promoting the proliferation of *Bifidobacterium* [3]. In addition, AOS can promote the growth of crop roots, while polysaccharides did not show a corresponding effect [4].

As one of the modifying enzymes of alginate, alginate is degraded to low molecular oligosaccharides by alginate lyase by a β-elimination mechanism. Alginate lyases are mainly derived from marine bacteria (*Pseudomonas*, *Vibrio*), soil bacteria (*Klebsiella*, *Azotobacter*), viruses (*Chlorella vius*), fungi (*Corollospora intermedia*) and other microorganisms; marine algae (*Laminaria japonica*); marine echinoderms and mollusks (*Haliotis discus hannai*) [5]. According to the CAzy database, alginate lyases are classified into 14 polysaccharide lyase (PL) families, including PL5, PL6, PL7, PL8, PL14, PL15, PL17, PL18, PL31, PL32, PL34, PL36, PL39 and PL41 families. In addition, alginate lyases can be classified into polyG-lyase (EC4.2.2.11), polyM-lyase (EC4.2.2.3) and bifunctional lyase because of the difference in substrate specificity [6]. According to the action mode, endo-alginate lyase degrades the substrate to oligosaccharides with a degree of polymerization of 2 or more, while exo-alginate degrades the substrate to monosaccharides. In recent years, alginate lyases with both endo- and exo-activities have been gradually characterized, and their simple and clear product distributions have attracted extensive attention in tailoring special AOS [7]. However, the commercial application of alginate lyases is largely limited by low activity and poor adaptability to industrial environments [8]. Most of the alginate lyases with mixed endo/exo-mode belong to the PL17 family, and only Alg2951 derived from *Alteromonas portus* HB161718^T^ belongs to the PL7 family. To meet the environmental conditions for industrial applications, the discovery of extreme alginate lyases such as thermophilic and salt-tolerant enzymes have received extensive attention. Among them, cold-adapted enzymes have gradually attracted widespread attention in industrial applications [9]. Biocatalysis at lower temperatures reduces the instability of some biologically active substances. Therefore, with the biocatalytic degradation process of alginate from brown-algae at lower temperatures, more active substances can be retained [10]. The use of alginate lyases with high activity and catalytic efficiency at low temperature can reduce energy consumption and cost in the process of producing bioethanol from brown algae crops. The inactivation of mesophilic and hyperthermic enzymes requires higher temperatures and consumes more energy. Low temperature cannot only effectively prevent the reproduction of harmful microorganisms, but also selectively inactivate the alginate lyase by raising the temperature slightly to achieve the purpose of terminating the reaction [9,11]. Alginate lyases with excellent biochemical properties at low temperature have potential in industrial applications such as food, medicine and biotechnology.

The cold-adapted alginate lyases found so far are concentrated in the PL6, PL7, PL15 and PL17 families [7,12,13,14]. However, most of these characterized cold-adapted alginate lyases have good stability at low temperature, but lose more specific activity, and the optimal temperature is not lower than 20 °C. In relation to the PL7 family, there are dozens of alginate lyases that have been biochemically characterized, including some structural resolved, such as AlyA5 isolated from *Z. galactanivorans* and VxAly7D isolated from *Vibrio xiamenensis* QY104 [15,16]. The alginate lyases of the PL7 family have broad substrate specificities, and different enzymes have different modes of action. However, it is difficult for the discovered enzymes to have both high activity at low temperature and cold-tolerance, such as Aly3C derived from *Psychromonas* sp. C-3, AlyL1 derived from *Agarivorans* sp. L11 [13,17].

Herein, a cold-tolerant and psychrophilic PL7 family alginate lyase (TAPL7A) from *Thalassotalea algicola* was firstly cloned and expressed in *Escherichia coli*. We investigated the enzymatic properties and product distribution of TAPL7A, in detail. Its optimal temperature is 15 °C and optimal pH is 8.0. This enzyme is the first PL7 family alginate lyase with high activity and stability below 0–30 °C. Furthermore, TAPL7A adopts a hybrid endo/exo-mode to degrade three substrates into oligosaccharides with degrees of polymerization of 1–2 at low temperature, with a preference for polyM. This work provides a new psychrophilic and cold-tolerant alginate lyase for the preparation of alginate oligosaccharides, and provides new data for the study of the structure and functional mechanism of psychrophilic enzymes.

## 2. Results

### 2.1. Sequence Analysis of Gene TAPL7A

**Figure 1:**
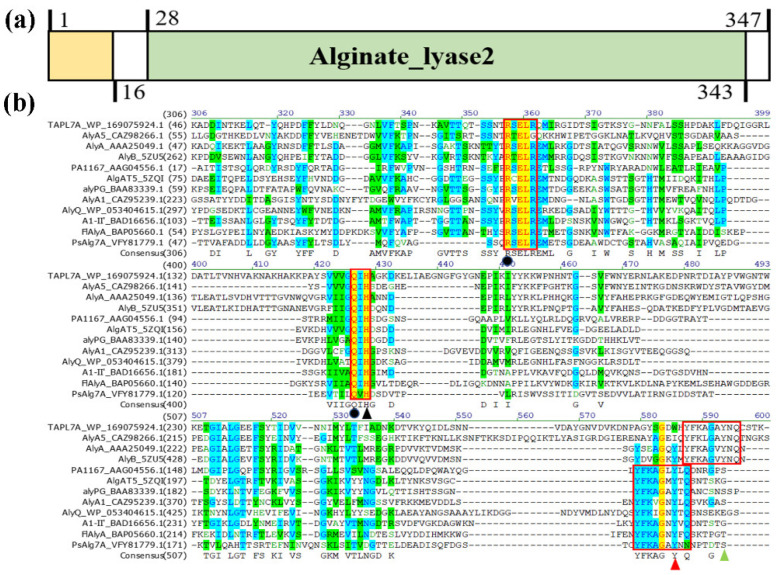
The domain analysis and multiple amino-acid sequence alignment of TAPL7A and other alginate lyases of PL7 family. (**a**) The domain of TAPL7A. (**b**) AlyA5 (CAZ98266.1) from *Zobellia galactanivorans* Dsij^T^. AlyA (AAA25049.1) from *Klebsiella pneumoniae subsp*. aerogenes, AlyB (5ZU5) from *Vibrio splendidus*, PA1167 (AAG04556.1) from *Pseudomonas aeruginosa* PAO1, AlgAT5 (5ZQI) from *Defluviitalea*, alyPG (BAA83339.1) from *Corynebacterium* sp. ALY-1, AlyA1 (CAZ95239.1) from *Zobellia galactanivorans* Dsij^T^, AlyQ (WP_053404615.1) from *Persicobacter* sp. CCB-QB2, A1-Ⅱ’ (BAD16656.1) from *Sphingomonas* sp. A1, FlAlyA (BAP05660.1) from *Sphingomonas* sp. A1, PsAlg7A (VFY81779.1) from *Paradendryphiella salina*. Shaded yellow indicates identical and similar amino-acid residues in alginate lyase. The red boxes indicate the positions of the three conserved regions. Black triangles are catalytic bases, red triangles and green triangles represent catalytic acids of PL7 lyase in different red boxes, respectively. Black circles indicate the neutralization residues.

We first cloned and analyzed the gene of alginate lyase TAPL7A from *Thalassotalea algicola* in this study. The open reading frame (ORF) of TAPL7A, consisting of 1041 bp of nucleotides, encoded a putative alginate lyase composed of 347 amino acids with a theoretical molecular mass of 36.76 kDa. The isoelectric point (*p*I) of TAPL7A is 6.17. TAPL7A containing a 16-residue signal peptide (Met1-Ala16). Based on the results of the conserved domain analysis, TAPL7A contains only an alginate_lyase2 domain of 214 amino acids (Phe28-Arg343) (Figure 1a). TAPL7A has the highest identity of 43% with AlyA5 from *Zobellia galactanivorans* (GenBank accession no. CAZ98266.1) [15], which indicates that TAPL7A is a new member of the PL7 family. In order to study the relationship between the amino-acid sequence and structure function of TAPL7A, 11 structurally resolved alginate lyases from the PL7 family were selected for sequence alignment (Figure 1b). It is evident that TAPL7A contains conserved regions such as “QIH”, “YFKAGVYNQ” and “(R/E) (S/T/N) EL”, which form the active center in PL7 family alginate lyases and play a role in substrate recognition and catalysis [13]. The two catalytic residues His, Tyr and two neutralization residues Arg, Gln predicted to be involved in the catalytic process based on sequence alignment were also conserved in PL7 alginate lyases.

**Figure 2:**
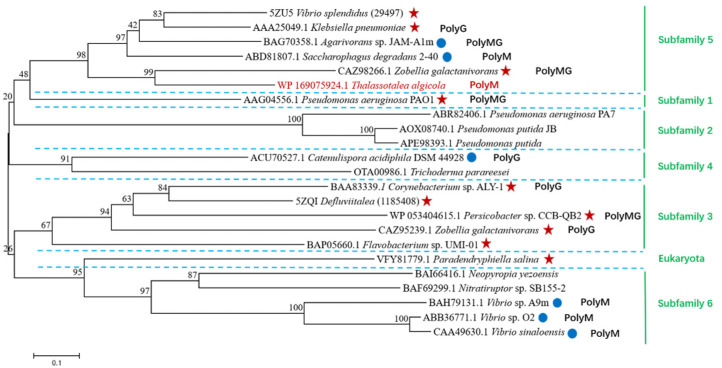
Phylogenetic analysis of TAPL7A with other alginate lyases of PL7 family. Subfamilies are separated by blue dotted lines according to the CAZy database. Enzymes for structure-solved are indicated by red five-pointed stars. Characterized enzymes are indicated by blue circles. PloyMG, PolyM, and PolyG represent the specific substrates of each enzyme, respectively. The red font is TAPL7A. The green vertical lines and fonts on the right represent the various subfamilies and PsAlg7A of eukaryotic origin.

According to the CAZy database, the PL7 family is subdivided into 5 subfamilies 1, 2, 3, 4, 5 based on sequence similarity. In recent years, some PL7 enzymes with low sequence similarity to other enzymes in the database have been classified into a new subfamily 6 [13]. To further identify the evolutionary relationship of TAPL7A, 12 sequences from 5 subfamilies, 1 eukaryotic origin and 5 unclassified sequences were selected to construct a phylogenetic tree according to the CAZy database. It was found that TAPL7A clusters with representative enzymes of subfamily 5 (Figure 2), thus TAPL7A is considered as a new member of the subfamily 5. The results are also consistent with sequence homology. Several alginate lyases have been characterized in subfamily 5, but their substrate specificity is not limited by the subfamily. For example, AlyA (GenBank accession no. AAA25049.1) from *Klebsiella pneumoniae subsp*. *aerogenes* prefers polyG [18], AlyA5 (GenBank accession no. CAZ98266.1) from *Zobellia galactanivorans* Dsij^T^ prefers polyMG [15], and Alg7D (GenBank accession no. ABD81807.1) from *Saccharophagus degradans* 2–40 prefers polyM [19]. Therefore, the substrate specificity of TAPL7A cannot be predicted based on sequence alignment and phylogenetic-tree analysis alone, although it shares the highest identity with AlyA5.

### 2.2. Cloning and Expression of TAPL7A

**Figure 3:**
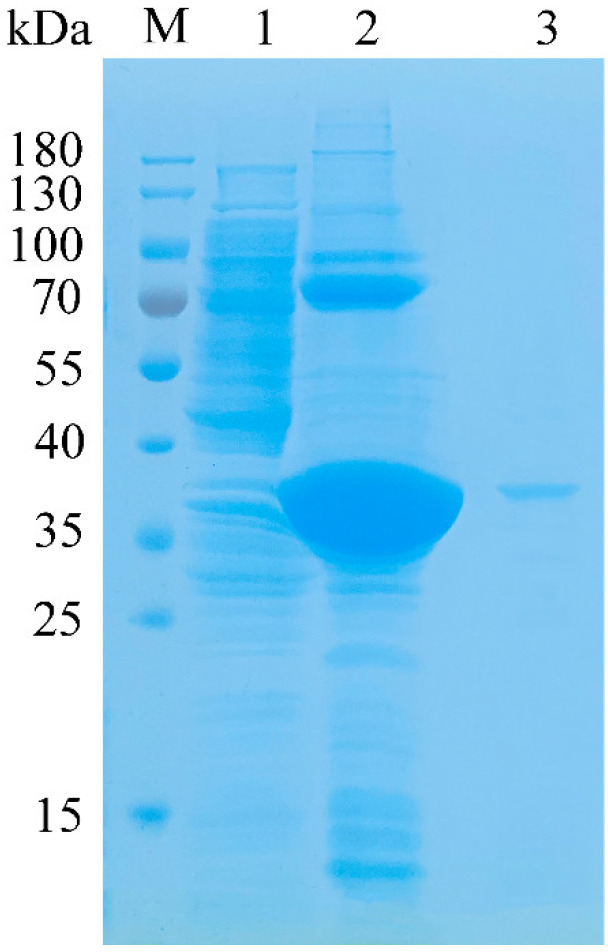
SDS-PAGE analysis of the molecular mass and purification effect of TAPL7A. Lane M protein: restrained marker (Thermo Scientific, USA); lane 1: the supernatant of *E. coli*-pET21a-TAPL7A; lane 2: induced cell lysate of *E. coli*-pET21a-TAPL7A; lane 3: renatured purified TAPL7A.

After heterologous expression, most of the recombinant proteins aggregated in the sonicated bacterial pellet in the form of body proteins (Figure 3, Line 1 and line 2). After renaturation of the body proteins with reference to the method of Singh et al. [20], the active crude enzyme solution was obtained. Recombinant TAPL7A was purified by Ni-NTA Sepharose affinity chromatography. The target protein expression and purification were verified by SDS-PAGE analysis. As shown in Figure 3 (Line 3), it can be observed that the band of the target protein TAPL7A is clearly located between 35 kDa and 40 kDa, which is consistent with the predicted molecular mass of 36.76 kDa. The molecular mass of PL7 family alginate lyases mainly depend on their modular domains. The molecular weight of single-domain PL7 lyases is concentrated between 25–40 kDa, such as AlyA5 [15]. There are also alginate lyases of the PL7 family that contain, in addition to the catalytic domain, one or two carbohydrate-binding domains (CBMs) involved in substrate binding and regulation of product distribution [21,22].

### 2.3. Substrate Specificity and Enzymatic Kinetics of TAPL7A

Three substrates (0.5% *w*/*v* sodium alginate, 0.5% *w*/*v* poly M and 0.5% *w*/*v* poly G) were used to perform activity assays and determine the substrate specificity of TAPL7A. The recombinant TAPL7A showed higher activity towards polyM (4186.1 U/mg) than that to polyG (2494.8 U/mg) and sodium alginate (2314.9 U/mg) (Table 1). Thus, TAPL7A is a new PL7 alginate lyase with a preference for polyM and can degrade polyG and sodium alginate with the same activity.

The kinetic parameters (*K*_m_ and *V*_max_) of TAPL7A towards polyM, polyG and sodium alginate were calculated according to hyperbolic regression analysis as shown in Table 1. The *K*_m_ values of TAPL7A towards polyM, polyG, and sodium alginate were 3.43 mM, 1.89 mM and 0.26 mM, respectively. TAPL7A is considered to have the strongest affinity for the M-M blocks. It suggested that TAPL7A exhibited the higher catalytic efficiency towards G-block than that towards M-block and MG-block. Although the activities of TAPL7A on polyG and sodium alginate are only half of that on polyM, the catalytic efficiency are higher. It is speculated that the isoleucine (I) in the conserved motif “QIH” residue recognizes substrates, and that alginate lyases containing the “QIH” motif generally show a preference for polyG or polyMG blocks [23], similar to AlyA5, which has the highest identity to TAPL7A, and is a polyMG-preferred enzyme [15].

### 2.4. Effect of Temperature and pH on TAPL7A

**Figure 4:**
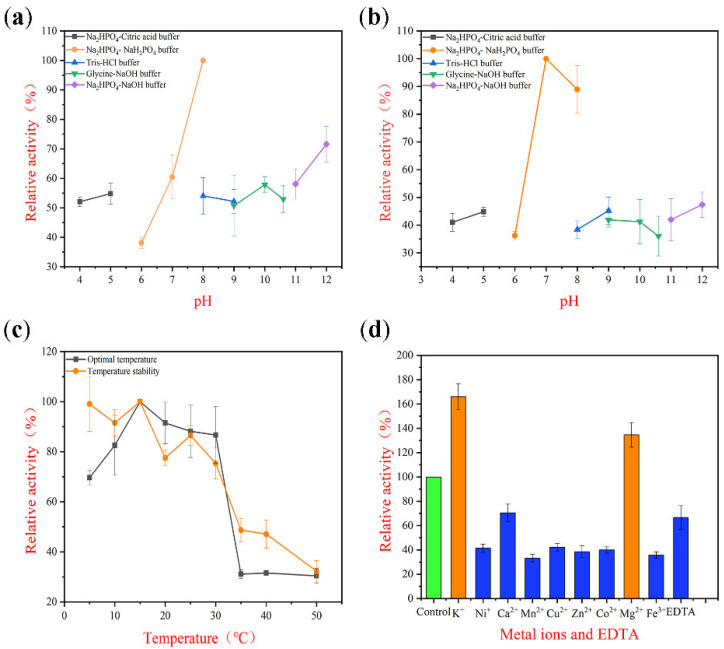
Biochemical characterization of TAPL7A. (**a**) The optimal pH of TAPL7A. (**b**) The pH stability of TAPL7A. (**c**) The optimal temperature and temperature stability of TAPL7A. (**d**) The effects of metal ions and EDTA on activity of TAPL7A. Orange represents metal ion activation; blue represents metal ion inhibition.

TAPL7A exhibits surprising psychrophilicity, showing maximum activity at 15 °C and retaining more than 80% of its maximum activity between 5–30 °C (Figure 4c). In addition, the optimal incubation temperature for TAPL7A was 15 °C, and more than 70% of the residual activity was maintained after incubation at 5–30 °C for 1 h (Figure 4c). In general, the optimal temperature below 35 °C and maintaining more than 50% of the maximum activity at 20 °C can be referred to as cold-adapted enzymes (Table 2). Several cold-adapted alginate lyases have been reported with optimal temperatures no lower than 20 °C [13,17,23,24,25,26]. This psychrophilic and highly cold-tolerant property of TAPL7A is rare among the cold-adapted alginate lyases. To the best of our knowledge, TAPL7A is currently the alginate lyase with the lowest optimal temperature 15 °C [27]. In order to highlight the excellent cold adaptation of TAPL7A, several cold-adapted enzymes were compared (Table 2). Characterized, cold-adapted alginate lyases exhibit either high activity at low temperature with poor cold tolerance, or strong cold tolerance with low activity at low temperature. For example, AlyL1 derived from *Agarivorans* sp. L11 has better cold resistance, but when the temperature is lower than 15 °C, its activity is only 20–50% of the maximum activity [28]. The thermophilic mechanism of thermophilic enzymes is currently thought to be their more compact conformation and rigid structure (such as disulfide bonds) [29]. Compared to thermostable enzymes, cold-adapted enzymes have highly flexible structural features. The high flexibility also comes with a stability trade-off, leading to thermal instability and, in the case of a few studies, cold instability [9,11]. The possible relatively loose structure of TAPL7A explains its low activity and instability in the environment at temperatures above 30 °C (Figure 4c). It is evident that compared to other cold-adapted enzymes, TAPL7A has more potential for industrial applications in low temperature environments (Table 2).

The optimal pH of TAPL7A is 8.0 (Figure 4a). Furthermore, TAPL7A maintained more than 50% of the maximum activity at pH 4.0–5.0 and pH 7.0–12.0, and maintained a high residual activity after incubation at the same pH environment (Figure 4a). The enzyme was mostly stable at pH 7.0–8.0 (Figure 4b). Most of the cold-adapted alginate lyases maintain high activity in the pH range near neutral, but their activity decreases sharply in the acidic and strongly alkaline range. For example, the enzymatic activity and stability of TsAly7B dropped below 40% of the maximum activity in environments below pH 6.0 and above pH 9.0 [22]. In contrast, TAPL7A has good pH adaptability.

### 2.5. Effect of Metal Ions on TAPL7A

The effect of metal ions on enzyme activity was also investigated (Figure 4d). K^+^ and Mg^2+^ can activate the activity of TAPL7A, while Ni^+^, Ca^2+^, Mn^2+^, Zn^2+^, Cu^2+^, Co^2+^ and Fe^3+^ inhibit the activity of TAPL7A. In marine environments, the activity of alginate lyases including TAPL7A are normally activated by K^+^ and Na^+^, such as AlgNJ04 from *Vibrio* sp. NJ-04 [30]. Both divalent/trivalent metal ions other than Mg^2+^ and the metal ion chelator EDTA inhibited the activity of TAPL7A. The effect of metal ions on TAPL7A activity is similar to that of Alg2951 from *Alteromonas portus* HB161718^T^ [31].

### 2.6. Effect of NaCl on TAPL7A

**Figure 5:**
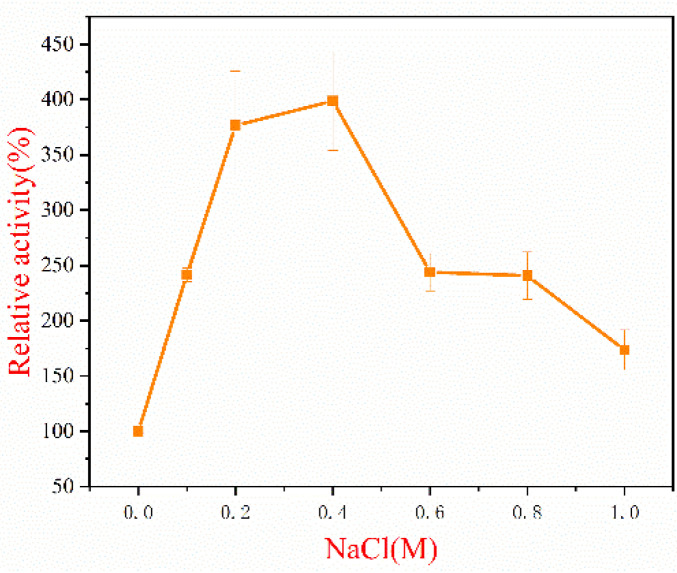
Effect of NaCl on TAPL7A.

Adapted to the marine environment, many alginate lyase enzymes are salt tolerance or/and salt activated [24,26,34]. For instance, the activity of AlgM4 from *Vibrio weizhoudaoensis* M0101 was significantly enhanced approximately 7-folds by 1.0 M NaCl [34]. Salt tolerance of TAPL7A was observed at NaCl concentrations of 0.1–1.0 M, taking the activity at 0 M NaCl as 100%. As shown in Figure 5, recombinant TAPL7A showed activity at 0 M NaCl, while increasing concentrations of NaCl (0–1.0 M) exhibited an increase and then a decrease in activity. The enzyme activity was increased 3-folds in the presence of 0.4 M NaCl. The activity of TAPL7A increased rapidly at NaCl concentrations in the range of 0–0.4 M and started to decrease slowly at concentrations above 0.4 M. Thus, TAPL7A is a new salt-activated alginate lyase. An amount of 0.4 M NaCl is comparable to the salt concentration of the optimal growth environment of *Thalassotalea algicola* [35]. The activity of TAPL7A at 1 M NaCl concentration was still higher than 0 M, which may indicate that its adaptation to salt concentration fluctuations varies with the salt concentration of the original growth environment of the strain. Similarly, AlyC3 derived from *Psychromonas* sp. C-3 has similar adaptations to environmental salt concentrations [13]. Several salt-activated alginate lyases have been found to have different mechanisms of salt tolerance. The salt activation of AlyPM derived from *Pseudoalteromonas* sp. SM0524 is due to its affinity for the substrate being enhanced by NaCl [26], while the salt activation of AlgM4 is due to the fact that its secondary structure can be altered by NaCl, possibly enhancing its substrate affinity and resistance to thermal denaturation [34]. The salt activation mechanism of TAPL7A requires further study.

### 2.7. Products Distribution and Action Pattern of TAPL7A

**Figure 6:**
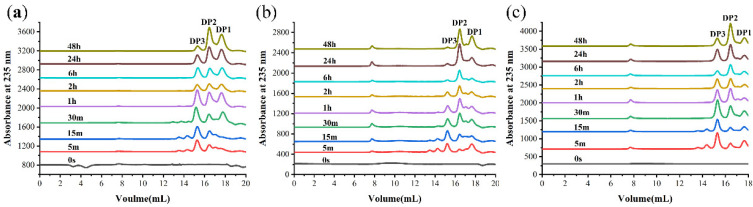
Analysis of 0–48 h products of TAPL7A by FPLC with (**a**) sodium alginate, (**b**) polyM, (**c**) polyG. The eluents were detected by measuring the absorbance at 235 nm.

**Figure 7:**
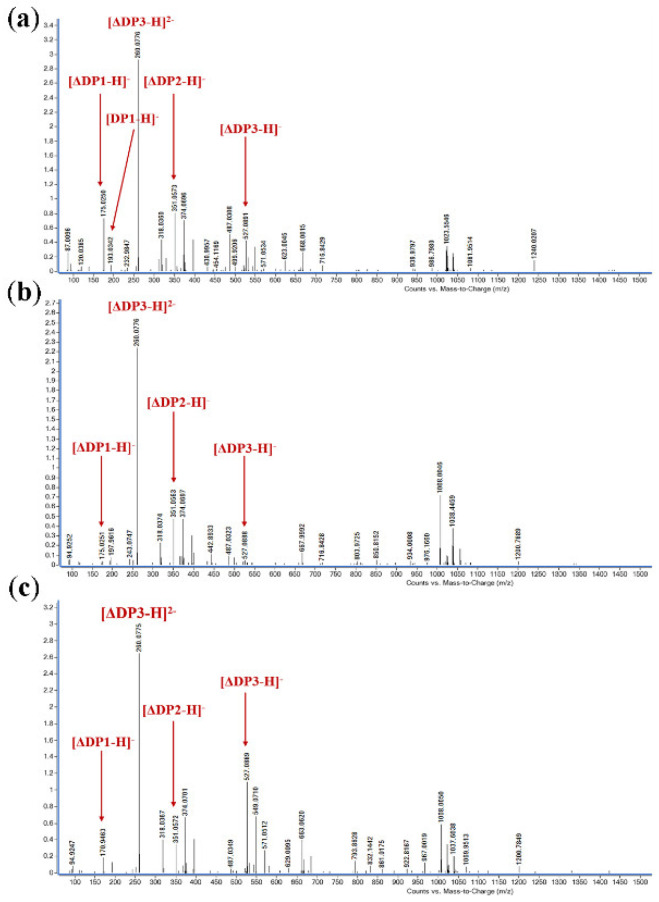
Analysis of 48 h product composition of TAPL7A by ESI-MS with (**a**) sodium alginate, (**b**) polyM, (**c**) polyG as substrates; The oligo-uronates are commonly described by their degree of polymerization (DPx). Oligo-uronates with the unsaturated terminal uronate are denoted in this study as ΔDPx, and such an oligomer would be ΔDP1.

The degradation products of TAPL7A were analyzed by FPLC over a time gradient of 0–48 h (Figure 6). In the early stage of the reaction, the three substrates were mainly degraded to trisaccharides by TAPL7A. As the reaction progresses, trisaccharides are gradually degraded into disaccharides and monosaccharides. The verification of the composition of the degradation products was undertaken using ESI-MS (Figure 7). When sodium alginate, polyM and polyG are used as substrates, oligomers of DP1, 2 and 3 (Signals of 175.02 *m*/*z* [ΔDP1-H]^−^, 351.05 *m*/*z* [ΔDP2-H]^−^, and 527.01 *m*/*z* [ΔDP3-H]^−^) are released as end products (Figure 7a–c). Consistent with the FPLC results, a signal of the monosaccharide conversion product 4-deoxy-ʟ-erythron-5-hexoseuloseuronate acid (DEH) was also detected in the ESI-MS spectrum. Therefore, in the reaction process of TAPL7A, endo-activity is mainly used in the early stage, and exo-activity is mainly used in the later stage. It can be seen from the above results that TAPL7A can efficiently degrade three substrates into monosaccharide and disaccharide with a mixed endo/exo-mode. The product distribution of endo-lyase of the PL7-5 subfamily is concentrated in DP2-5, while the exo-lyase is concentrated in DP1 and 2. For example, endo-lyase AlyL2 derived from *Agarivorans* sp. L11 [28] and exo-lyase AlyA5 derived from *Zobellia galactanivorans* [15].

AOS is an excellent material for food, medicine and agricultural applications, and the difference in its degree of polymerization and structural composition will also lead to different effects. The unsaturated monosaccharide 4-deoxy-ʟ-erythron-5-hexoseuloseuronate acid (DEH) produced by exo-lyases can be non-enzymatically converted to KDG for bioethanol production [36]. The AOS produced by the degradation of substrates by Alg2951 with the same endo/exo-mode of action has excellent antioxidant capacity [31]. Therefore, TAPL7A is an excellent tool enzyme for the production of high value-added AOS with low energy consumption and acid-base stability.

### 2.8. Molecular Modeling

**Figure 8:**
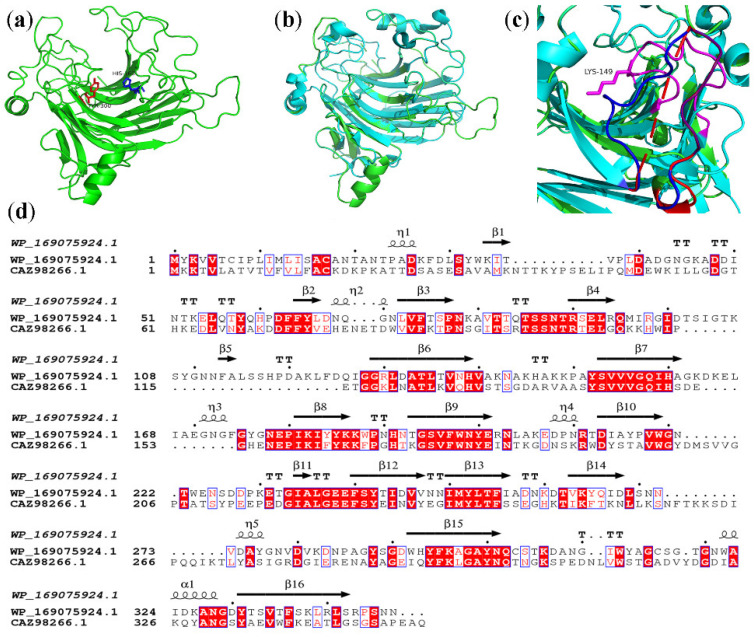
(**a**) Overall structure of TAPL7A; (**b**) The structural comparison of TAPL7A (green) and AlyA5 (cyan); (**c**) Comparison of loops at the ends of catalytic groove of TAPL7A (red and purple) and AlyA5 (blue); (**d**) Structure-based sequence alignment of TAPL7A and AlyA5. Conservative amino acids are highlighted by the red background and similar amino acids are highlighted by the red letters. α-helices are displayed as squiggles, β-strands are rendered as arrows, strict β-turns as TT letters. This figure has been generated using the program ESPRIPT 3.0.

Most of the characterized PL7 family alginate lyases are endo-lyase with a wide range of substrate specificities. The mode of action and substrate preference of enzymes of the PL7 family are complex, such as the bifunctional endo-lyase PA1167 [37], the bifunctional exo-lyase VxAly7D [16], and the mixed endo/exo Alg2951 [31]. Therefore, structural studies of the PL7 family provide useful information on the mode of substrate recognition and depolymerization. The structure of the alginate lyase AlyA5 derived from *Zobellia galactanivorans* (PDB ID: 4BE3) was used as a template to construct a structural model of TAPL7A with 100% confidence using the online URL PHYRE2 (Figure 8a). The overall structure of TAPL7A is typical of the PL7 β-sandwich jelly roll-fold [15]. TAPL7A has 16 anti-parallel β-strands to form the main structure, of which three conserved motifs “(R/E) (S/T/N) EL”, “QIH” and “YFKAGVYNQ” are located at β-4, β-7 and β-15, respectively. These three chains make up the catalytic cavity. Catalytic residues His160 and Tyr300 of TAPL7A predicted based on sequence alignment are displayed in the homology model (Figure 8a). The relatively loose structure of TAPL7A and the greater opening of the flexible loops can be seen from the structural alignment of TAPL7A (green structure) with AlyA5 (blue structure) (Figure 8b). This may also be evidence of TAPL7A psychrophilic and cold tolerance.

Most of the PL7 family lyases have multiple flexible loops in their domains, which play a role in structural stability and mechanism of action. The structural determinants responsible for the exolytic activity of AlyA5 are three large additional loops, Trp197-Asp217, Ser257-Glu284 and Gly304-Asp318, which close the catalytic groove of the concave β-sheet at one end (Figure 8b) [15]. The structural basis of polyM-specific FlAlyA indicates that two flexible loops linked by hydrogen bonds above its catalytic cleft are involved in controlling the substrate specificity [38]. Comparing the structures of FlAlyA and AlyA5, it can be found that there are two to three flexible loops above their active clefts that play a role in substrate binding. However, the active cleft end of FlAlyA is unblocked, unlike AlyA5, which is blocked by three flexible loops. Structural alignment indicated that the loop blocked at the end of the catalytic groove in AlyA5 (blue loop Trp197-Asp217 in Figure 8c,d) was missing 7 amino acids at the corresponding position in TAPL7A (red putative loop Trp219-Thr232 in Figure 8c,d) and could not form a loop, so that the catalytic groove of TAPL7A was opened. As for one of the possible reasons for the exo-lyase activity, the loop His139-Ser153 in TAPL7A that extends into the catalytic groove but does not block the catalytic groove (purple loop Figure 8c,d). Only Tyr152 and Ser153 of this loop are conserved in AlyA5, but the remaining positions are replaced by other residues (His139-Ala151). In particular, the hydrophobic Ala138 at the top of this loop in AlyA5 is replaced by a basic Lys149 in TAPL7A, which may form hydrogen bonds with the substrate. Further research is needed, and structural elucidation will help to understand how the hybrid mode of action works.

## 3. Discussion

The ocean, which makes up 71% of the surface of the world, is a veritable treasure trove. In this treasure trove, marine enzymes represent an essential resource. Marine enzymes may have different characteristics from enzymes of terrestrial origin and show excellent potential applications in biological and industrial technologies. The special environment of the ocean may create special enzymes adapted to the industrial environment, such as heat-, salt- and base-tolerant enzymes. The potential of marine enzymes for biotechnology and industrial applications has been shown in numerous studies [39,40,41,42,43,44,45]. For example, thermostable proteases of marine origin have the potential to be used commercially in protein processing and in the production of protein hydrolysates in the detergent industry [46]. It is foreseeable that marine enzymes will become an important tool in various industrial fields in the future.

Alginate lyase is a key enzyme in the marine environment, which plays an important role in alginate degradation. Although hundreds of alginate lyases have been reported, only a few of them have specific properties. In this study, we report, for the first time, on both cold-adapted and cold-tolerant alginate lyase and their hybrid mode for efficient preparation of alginate oligosaccharides. TAPL7A is a new member of the PL7-5 subfamily and shares the highest homology of 43% with the alginate lyase AlyA5. TAPL7A has a typical PL7 family β-sandwich jelly roll-fold. The conserved motifs “QIH”, “YFKAGVYNQ” and “(R/E) (S/T/N) EL” of PL7 family alginate lyases may play a catalytic and substrate binding role in TAPL7A. TAPL7A has an optimal pH of 8.0 and also has a wide range of pH adaptability and pH stability. The enzyme maintains 80–100% activity between 5–30 °C. Moreover, 70–100% of the activity can be retained after incubation at 5–30 °C for 1 h. To the best of our knowledge, this enzyme has the lowest optimal temperature (15 °C) and better cold adaptability among the alginate lyases found so far. In the presence of 0.4 M NaCl in the solution system, the activity of TAPL7A increased 3-fold compared with the absence of NaCl. Meanwhile, 0.4 M (2% *w*/*v*) NaCl is also the optimal salt concentration for *Thalassotalea algicola* growth [35]. The salt concentration-activated mechanism of TAPL7A may be adapted to the changes in the growth environment of *Thalassotalea algicola*. K^+^ and Mg^2+^ activate the activity of TAPL7A, while Ni^+^, Ca^2+^, Mn^2+^, Zn^2+^, Cu^2+^, Co^2+^ and Fe^3+^ inhibit its activity.

In addition, the enzyme has a unique hybrid endo/exo-mode. TAPL7A preferentially degrades polyM, but also has half the degradation activity of polyM for alginate and polyG. Early in the reaction, the three substrates can be degraded into alginate oligosaccharides with a degree of polymerization of DP1-3, and the trisaccharides can be degraded into monosaccharides and disaccharides with the progress of the reaction. Although we do not have the detailed crystal structure of TAPL7A, based on homologous model and sequence alignment, we speculate that one of the factors affecting its unique degradation pattern is the flexible loops (His139-Ser153) above its catalytic cleft. For example, Qin et al. studied the structural basis of the alginate lyase FlAlyA and confirmed that the flexible loops near the active cleft of the PL7 family lyase have an important impact on substrate binding and mode of action [38]. In regard to the excellent psychrophilic and cold tolerance of TAPL7A, we speculate that it is due to its relatively loose overall structure. A more in-depth study of TAPL7A, especially the structural basis, will provide a solid foundation for the analysis and understanding of cold-adaptive mechanisms and endo/exolytic patterns. With the growing demand for high value-added alginate oligosaccharides, alginate lyases with their special degradation mechanism and product distribution will become the first choice. At the same time, psychrophilic and cold-tolerant alginate lyases will consume less capacity during the production of alginate oligosaccharides, reducing costs and retaining more biological activity. In conclusion, TAPL7A is a potentially promising tool for tailoring alginate oligosaccharides at low temperature.

## 4. Materials and Methods

### 4.1. Materials

Sodium alginate was provided by Sigma-Aldrich (M/G ratio 77/23 isolated from *Macrosystis pyrifera*, viscosity ≥ 2000 Cp, St. Louis, MO, USA). PolyM and polyG with average DPs of 39 (purity: about 95%; M/G ratio: 3/97 and 97/3; average molecular weight: 7200 Da) were purchased from Qingdao BZ Oligo Biotech Co., Ltd. (Qingdao, China). All other chemicals and reagents were of analytical grade. In this study, *E. coli* DH5α was used as the host for plasmid construction and *E. coli* BL21 (DE3) was used as the host for gene expression. E. coli was incubated at 37 °C in Luria–Bertani (LB) broth or LB broth agar plates (LB broth supplemented with 1.5% *w*/*v* agar) that both contained 100 μg/mL ampicillin.

### 4.2. Sequence Analysis of Gene TAPL7A

The theoretical molecular weight (Mw) and isoelectric point (*p*I) of TAPL7A were calculated online at the online website Compute *p*I/Mw tool of Expasy (https://web.expasy.org/compute_pi/, accessed on 10 September 2021). Signal peptides predicted by the online website SignalP-6.0 (https://services.healthtech.dtu.dk/service.php?SignalP-6.0, accessed on 23 September 2021). The alignment of protein sequences of TAPL7A and other enzymes was performed with ESPript program (https://espript.ibcp.fr/ESPript/ESPript/index.php, accessed on 29 September 2021). The conserved domains were analyzed by Simple Modular Architecture Research Tool (SMART) (http://www.ebi.ac.uk/Tools/pfa/iprscan/, accessed on 15 October 2021) [47]. According to the protein sequences of PL7 family enzymes, the phylogenetic tree was constructed through the Molecular Evolutionary Genetics Analysis (MEGA) Program version 5.05. 

### 4.3. Cloning, Expression and Purification of TAPL7A

According to the genomic sequence information of *Thalassotalea algicola*, a pair of specific primers with *Nde* I and *Xho* I (TAPL7A-F: CGCCATATGTGCGCGAACACCGCGAACAC, TAPL7A-R: CCGCTCGAG TGAGCCGCCCGAGCAACAAC) was designed. The expression vector was pET-21a (+) plasmid and *E. coli* BL21 (DE3) was used as the expression host. The recombinant strain was incubated at 37 °C until OD600 was 0.4–0.6, and expression of the recombinant enzyme was induced at 22 °C for 36 h after the addition of 0.1 mM IPTG. Referring to the method of Singh et al., 8 M urea was used to dissolve the inclusion body proteins in the precipitate of the disrupted bacterial solution, and the renatured body proteins were obtained by dialysis [20,48]. According to the method of zhu et al., the renatured TAPL7A was purified by a Ni-NTA Sepharose column (GE Healthcare, Uppsala, Sweden), and the TAPL7A before and after purification was verified by 12% (*w*/*v*) sodium dodecyl sulfate polyacrylamide gel electrophoresis (SDS-PAGE) [49]. Protein concentration was determined by a protein quantitative analysis kit (Beyotime Institute of Biotechnology, Nantong, China).

### 4.4. Substrate Specificity and Enzymatic Kinetics of TAPL7A

Purified TAPL7A (50 μL) was reacted with 150 μL of each of the three substrates (sodium alginate, polyM and polyG) at 0.5% (*w*/*v*) for 30 min at 15 °C, followed by a boiling water bath for 10 min to terminate the reaction. One unit of enzymatic activity was defined as the amounts of enzyme required to increase absorbance at 235 nm (extinction coefficient: 6150 M^−1^·cm^−1^) by 0.01 per min. Substrate specificity was determined by the activity of TAPL7A in response to three different substrates (0.5% *w*/*v* sodium alginate, 0.5% *w*/*v* polyM and 0.5% *w*/*v* polyG). The kinetic parameters of the TAPL7A towards sodium alginate, polyM, and polyG were evaluated by measuring the enzyme activity with substrate at different concentrations as described previously [49]. The Lineweaver–Burk plots were used to calculate the kinetic parameters *K*_m_ and *V*_max_ based on the reaction rates of the enzymes with different concentrations of the three substrates (0.2–10.0 mg/mL) at 235 nm. The ratio of *V*_max_ versus enzyme concentration ([E]) was used to calculate the turnover number (*k*_cat_) of the enzyme. All experiments were performed with three replicates.

### 4.5. Effect of Temperature and pH on TAPL7A

The reactions were performed at 5–50 °C to investigate the optimal temperature for TAPL7A. TAPL7A was incubated at 5–50 °C for 1 h and then reacted with alginate at 15 °C for 30 min to evaluate its temperature stability. Purified TAPL7A and substrate were mixed in a 1:3 ratio in buffers of different pH values (4.0–12.0) and reacted at 15 °C for 30 min, and the activity was measured under the following standard conditions. In detail, the purified enzyme was mixed with the substrate in buffers with different pHs, which were 50 mM phosphate-citrate (pH 4.0–5.0), 50 mM NaH_2_PO_4_-Na_2_HPO_4_ (pH 6.0–8.0), 50 mM Tris-HCl (pH 7.0–9.0), 50 mM glycine-NaOH (pH 9.0–10.0), and 50 mM Na_2_HPO_4_-NaOH at 15 °C for 30 min. After incubation of TAPL7A in buffers of different pH values (4.0–12.0) for 24 h (4 °C), the residual activity of TAPL7A was assayed after 30 min of reaction with the substrate at the optimal temperature to assess the pH stability.

### 4.6. Effect of Metal Ions on TAPL7A

TAPL7A was incubated with various metal compounds and EDTA at a final concentration of 1 mM (50 mM Tris-HCl buffer, pH 8.0) at 4 °C for 24 h. The effect of metal ions was evaluated by detecting the participation of the enzyme after 30 min of reaction at 15 °C. In addition, the activity of the reaction mixture without any metal ion was regarded as control (100% relative activity). In order to improve the credibility of the experimental data, all experiments were performed with three replicates.

### 4.7. Effect of NaCl on TAPL7A

The salt tolerance of TAPL7A was observed at NaCl concentrations of 0–1.0 M. The reactions were performed at 15 °C. In addition, the activity of the reaction mixture without any NaCl was regarded as control (100% relative activity). In order to improve the credibility of the experimental data, all experiments were performed with three replicates.

### 4.8. Products Distribution and Action Pattern of TAPL7A

FPLC with Superdex peptide 10/300 GE Colum (GE Health) at 235 nm was used to separate and monitor the products. The reaction mixtures (400 μL) containing 100 μL of purified TAPL7A, 300 μL of substrates (0.5% *w*/*v* sodium alginate, 0.5% *w*/*v* polyM, and 0.5% *w*/*v* polyG, respectively) were incubated at 15 °C for 0–48 h. The samples were taken after reaction for 0 min, 5 min, 15 min, 30 min, 1 h, 2 h, 6 h, 24 h, and 48 h, respectively. The mixture was eluted with 0.2 M NH_4_HCO_3_ at flow rate of 0.8 mL/min. In addition, the final degradation products were identified by ESI-MS in negative ion mode, and the specific conditions of ESI-MS were as follows: ion source voltage, 4.5 kV; capillary temperature, 275–300 °C; tube lens, 250 V; sheath gas, 30 arbitrary units (AU); and the scanning mass range, 150–2000 *m*/*z*.

### 4.9. Molecular Modeling

The structure of alginate lyase AlyA5 (PDB ID: 4B3E) from *Zobellia galactanivorans* was used as the homology template, and the online software PHYRE 2.0 (http://www.sbg.bio.ic.ac.uk/phyre/html/page.cgi?id=index, accessed on 10 November 2021) was used to successfully construct the structural model of TAPL7A with 100% confidence.

## Figures and Tables

**Table 1:** Substrate specificity and kinetics of TAPL7A.

Substrate	Activity	*K*_m_ (mM)	*V*_max_ (μmol/s)	*k*_cat_ (s^−1^)
Alginate	2314.9 U/mg	0.26	0.0648	14.305
PolyM	4186.1 U/mg	3.43	0.0622	13.730
PolyG	2494.8 U/mg	1.89	0.07412	16.362

**Table 2:** Properties of TAPL7A and partially characterized cold-adapted alginate lyases.

Enzyme	PL Family	Organism	Optimal Temperature (°C)/pH	Activity/Residual Activity at 5–15 °C	Degree of Polymerization of the Product (DP)	Mode of Action	Refs.
TAPL7A	PL7	*Thalassotalea algicola*	15/8.0	80–100%/90–100%	1–2	Endo+Exo	This study
Alyw201	PL7	*Vibrio* sp. W2	35/8.0	No data-60%/No data-90%	2–6	Endo	[23]
AlyC3	PL7	*Psychromonas* sp. C-3	20/8.0	58–92%/No data	1–3	Endo	[13]
AlyL1	PL7	*Agarivorans* sp. L11	40/8.6	20–54.5%/80–95%	2–3	Endo	[17]
TsAly7B	PL7	*Thalassomonas* sp. LD5	30/7.6	10–40%/60–80%	2–4	Endo	[22]
AlyPM	PL7	*Pseudoalteromonas* sp. SM0524	30/8.5	19–45%/90–100%	2–4	Endo	[26]
AlgB	PL7	*Vibrio* sp. W13	30/8.0	No data	2–5	Endo	[32]
A9mT	PL7	*Vibrio* sp. A9mT	30/7.5	No data/60–75%	No data	No data	[33]
Alg2951	PL7	*Alteromonas portus* HB161718^T^	25/8.0	40–70%/100%	1, 3	Endo+Exo	[31]
AlyGC	PL6	*Glaciecola chathamensis*	30/7.0	50–60%/No data	1	Exo	[12]
AlgSH17	PL17	*Microbulbifer* sp. SH-1	30/7.0	No data	1, 2–6	Endo+Exo	[7]

## Data Availability

Not applicable.
